# The Therapeutic Potential of Metformin in Neurodegenerative Diseases

**DOI:** 10.3389/fendo.2018.00400

**Published:** 2018-07-19

**Authors:** Carola Rotermund, Gerrit Machetanz, Julia C. Fitzgerald

**Affiliations:** ^1^German Centre for Neurodegenerative Diseases, Tübingen, Germany; ^2^Department of Neurodegenerative Diseases, Centre of Neurology and Hertie Institute for Clinical Brain Research, University of Tübingen, Tübingen, Germany

**Keywords:** metformin, neurodegeneration, diabetes, Parkinson's disease, Alzheimer's disease, aging, mitochondria

## Abstract

The search for treatments for neurodegenerative diseases is a major concern in light of today's aging population and an increasing burden on individuals, families, and society. Although great advances have been made in the last decades to understand the underlying genetic and biological cause of these diseases, only some symptomatic treatments are available. Metformin has long since been used to treat Type 2 Diabetes and has been shown to be beneficial in several other conditions. Metformin is well-tested *in vitro* and *in vivo* and an approved compound that targets diverse pathways including mitochondrial energy production and insulin signaling. There is growing evidence for the benefits of metformin to counteract age-related diseases such as cancer, cardiovascular disease, and neurodegenerative diseases. We will discuss evidence showing that certain neurodegenerative diseases and diabetes are explicitly linked and that metformin along with other diabetes drugs can reduce neurological symptoms in some patients and reduce disease phenotypes in animal and cell models. An interesting therapeutic factor might be how metformin is able to balance survival and death signaling in cells through pathways that are commonly associated with neurodegenerative diseases. In healthy neurons, these overarching signals keep energy metabolism, oxidative stress, and proteostasis in check, avoiding the dysfunction and neuronal death that defines neurodegenerative disease. We will discuss the biological mechanisms involved and the relevance of neuronal vulnerability and potential difficulties for future trials and development of therapies.

## Introduction

The evolution of genomics has greatly advanced our understanding of the genetic contribution to neurodegenerative diseases and provided an entry point for studying the biological cascades leading to neuronal degeneration. The growing research areas of bioinformatics and systems medicine have also opened up opportunities for better targeted treatments and individualized therapies. However, even for diseases such as Alzheimer's and Parkinson's disease, in which much progress has been made, a clear link between genetics, underlying pathological processes and the resulting clinical phenotype seldom exists. Neurodegenerative diseases are currently incurable, debilitating conditions caused by the progressive degeneration and death of nerve cells and their prevalence is rising in today's society (1).

**Figure 1:**
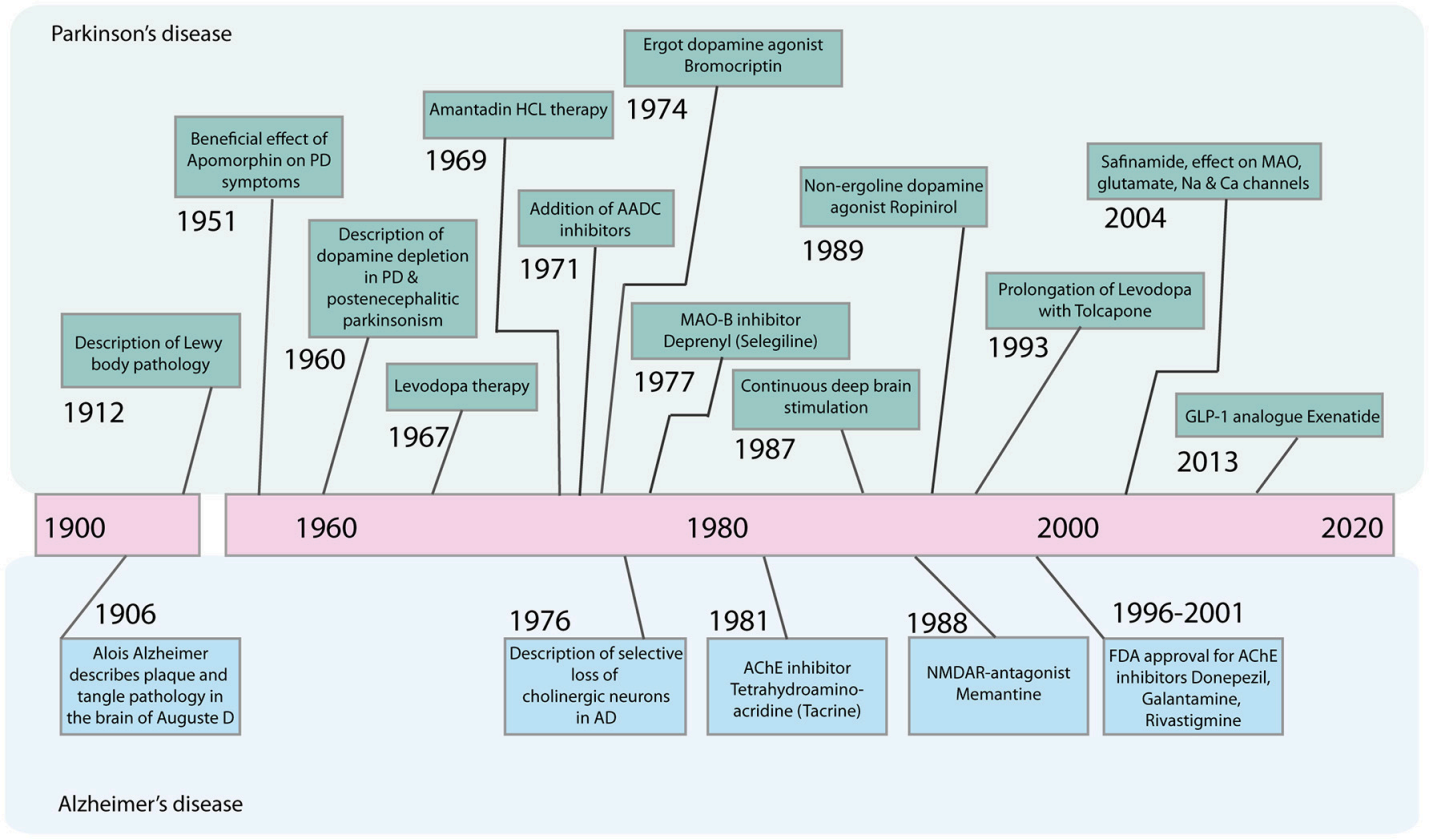
Timeline of major advances in the treatment of Parkinson's disease and Alzheimer's disease in the last century. AADC, Aromatic L-amino acid decarboxylase; AChE, Acetylcholinesterase; GLP-1, Glucagone-like Peptide 1; HCL, Hydrochloride; MAO-B, Monoamine oxidase B; NMDAR, N-Methyl-D-Aspartate Receptor.

Therefore, despite substantial advances in the development of symptomatic treatments for Alzheimer's disease (AD) and Parkinson's disease (PD) (Figure 1), there is still a major need for novel therapeutic strategies and disease-modifying treatments.

Given the complex and heterogeneous molecular basis of neurodegenerative disease the task can appear overwhelming and the previous decades have seen mostly disappointing clinical trial outcomes and subsequent lack of financial investment.

Gallega officinalis (French lilac) contains glucose-lowering guanidines and has been used for treatment of diabetes for centuries. The derivate metformin is a biguanide which was introduced in Europe in the 1950s and in the United States in the 1990s (2, 3). Metformin has recently been reported to decrease cardiovascular risk, restore ovarian function in polycystic ovary syndrome, reduce hepatic lipogenesis, fatty liver disease, and reduce oxidative stress (3, 4). The mechanisms by which metformin exerts its effects are still not fully defined but it is known that metformin inhibits glucose production in the liver and increases glucose uptake in peripheral tissues thereby lowering blood glucose levels (3, 5, 6). It is also accepted that metformin slows mitochondrial respiration via its direct action on complex I of the respiratory chain of mitochondria.

## The therapeutic potential of metformin: rationale

Metformin has the potential to interfere with neuronal longevity mechanisms and is therefore an interesting drug since it has already been approved for human use. However, human aging research in general has been slowed down by the lack of good aging models that can be used in the laboratory. Retaining aging signatures in reprogrammed neurons has been made possible by the use of direct reprogramming protocols (7, 8) but this may not be feasible for some research groups and time is needed for the technologies to be established in non-expert laboratories. New, simple and affordable methods to investigate the role of aging in human cells are still greatly needed.

Nonetheless, data from human and animal studies regardless of cell type have shown that dysregulation of insulin function contributes to aging and the development of neurodegenerative diseases (9). Insulin resistance and diabetes are increasingly recognized as a contributor to disease development especially in the field of dementias (10–12). Therefore, the rationale for using metformin is its potential to slow aging processes by acting on mitochondrial metabolism and insulin signaling. Slowing the aging process will be beneficial because quality of life could be improved in old age by delaying disease.

A link between diabetes and neurodegenerative diseases is for the most part accepted, although data is not unequivocal, and the exact mechanisms are unclear. A large body of data on metformin use in humans and animals with neurodegenerative diseases exists but metformin's therapeutic use is not yet accepted since the results are often conflicting. These different outcomes are dependent on disease, model system, species and the underlying biological pathways involved, which are now briefly reviewed.

## Dementia

Dementia is a common neurological disease of heterogeneous origin and the most important risk factor is aging. Dementia affects memory and other cognitive functions, interfering with a person's ability to carry out routine daily activities. According to the UN world population prospects, the number of persons aged 60 or over on the globe is estimated to grow approximately four times over the next 30 years (13) bringing the prediction that diagnoses of dementia will also rise. The most common form of dementia is AD but there are other types of dementia including vascular dementia, mixed dementia, frontotemporal dementia, dementia with Lewy bodies, and Parkinson's disease dementia.

### Alzheimer's disease

Alzheimer's disease (AD) is the most common neurodegenerative disease, with 45 million people worldwide affected (14). AD is characterized by progressive memory loss and decline of cognitive function.

Neurofibrillary tangles (NFTs, composed of abnormal tau protein) and amyloid plaques [composed of extracellular aggregates of amyloid-β (Aβ)] are pathological hallmarks of the AD brain (15–17). The NFT protein tau is associated with microtubules and is responsible for their stabilization (18). Tau pathology and synaptic loss correlates with cognitive impairments in AD patients (19). The amyloid plaque component Aβ derives from the sequential cleavage of the membrane protein APP (Amyloid precursor protein) by β-secretase BACE1 (β-site amyloid precursor protein cleaving enzyme 1) and the γ-secretase complex (20). Dysregulation, abnormal modification, and build-up of these protein structures in the brain are thought to be the major pathologies underlying AD.

From a genetic standpoint, most forms of AD are sporadic and of late onset but familial forms of early onset AD exist and are commonly caused by mutations in APP or presenilin (21–23). The underlying biological mechanisms leading to sporadic forms of AD have still not been defined. Inflammatory response, hormone regulation, mitochondrial dysfunction, and lysosomal dysfunction have been implicated, to name only a few processes. There is also growing genetic evidence for microglial involvement (24–26). Still, the main risk factors for developing AD are aging, genetic risk factors including being an APOE-ε4 allele carrier, variants in TREM2, and several GWAS loci, traumatic brain injury, cardiovascular risk factors, and several environmental risk factors (27–31).

### Diabetes and dementia: animal models

Most of the rodent models used to investigate the role of insulin and glucose metabolism in dementia have focused on AD. Insulin signaling and glucose tolerances are altered in APP/PS1 mice fed a high fat diet (32, 33), in partially leptin deficient (db/+)-APP/PS1 mice (34) and APP23-(ob/ob) mice (35). APP load may therefore boost susceptibility to disturbances of energy metabolism.

A high fat diet induces insulin resistance and promotes amyloidosis and memory impairment in both the Tg2576 mouse model of AD as well as in APP transgenic mice (36, 37). High fat diets or obesity could contribute to memory deficits even in wild type animals. However, some studies reviewed by Agusti and colleagues had no effect on cognition at all (38) leaving the topic still debated because of conflicting data. Diabetic rats show increased levels of APP, Aβ, and phosphorylated tau (39). These data suggest that alteration of energy metabolism via insulin signaling may contribute to Aβ generation and altered tau phosphorylation, two well know biochemical events associated with dementias. The modulation of insulin has been proven to be an effective strategy to protect neurons and synapses against toxic Aβ oligomers and to improve cognition in other AD animal models (40, 41). For example, glucagon-like peptide-1 (GLP-1), insulin-like growth factor 1 (IGF-1) as well as caloric restriction have all been shown to exert neuroprotective effects (42–44).

### Metformin and dementia: animal models

Until now only few animal studies have assessed the effect of metformin on cognitive decline and the results are not in line (Table 1). There are many different ways in which researchers can modulate energy metabolism in rodents to try to induce cognitive impairments and perhaps this has contributed to the variable data on metformin in this context. Some animals are fed high fat diets, others such as the (db/db) mice have a spontaneous mutation that cause them to be insulin resistant and obese. In three such high fat diet studies, metformin treatment reduces cognitive deficits (57, 58, 60), but one study found no improvement (59). In (db/db) mice, one study found metformin improved memory (53) whereas another study found no effect (55). It should be noted that in one study looking at normal aging in wild type mice, metformin had a detrimental effect on memory impairment (61). In this study activation of AMPK by phosphorylation was not measured and therefore it is not clear whether the metformin diet in these animals was optimal. More studies with proper controls are clearly needed to understand the effect of metformin in normal aging.

**Table 1:** Studies investigating the effect of metformin on neurodegeneration in rodent models.

**Study**	**Mouse strain**	**Disease model**	**G**	Starting age^*^	**Met dose + Application**	**Study/Met duration**	***n* per Group**	**Main findings**
**PARKINSON'S DISEASE**								
(45)	B6, Dat-Cre AMPKb1/2 KO	Day 20 + 22: MPTP: 30 mg/kg	M	8–10 weeks	100 mg/kg/day Drinking water	27 days	*n* = 6–10	Met decreases MPTP-induced loss of TH-positive neurons in ST but not SN and reduces astrogliosis Met induces AMPK phosphorylation in WT animals, although effects on TH-positive neurons are independent of AMPK-KO
(46)	C57BL	Day 1+2: MPTP: 10 mg	M	? 20–25 g	150 mg/kg/day Metal probe	7 days	*n* = 5	Met has no effect on MPTP-induced loss of TH-positive neurons in SN but reduces levels of microglia marker Iba1 Reduces amount of dopamine in the striatum
(47)	C57BL/6N	Day 7: MPTP: 15 mg/kg, 4x	M	8 weeks	200 or 400 mg/kg/day, Drinking water	14 days	*n* = 3 /10	Met reduces MPTP-induced loss of TH-positive neurons in SN Met induces pCreb and PGC1α in SN and ST
(48)	C57BL/6	Day 1–7: MPTP: 30 mg/kg	M	10 weeks 20–25 g	200 mg/kg/day Injection	14 days Met: day 8–14	*n* = 6	Met ameliorates MPTP-induced motoric deficits Met decreases MPTP-induced loss of TH-positive neurons in SN and reduces astrogliosis Met induces AMPK and AKT phosphorylation, reduces levels of phosphorylated mTor and induces BDNF in the SN
(49)	C57BL/6	5 weeks: every 3.5 day; MPTP: 20 mg/kg + 250 mg/kg probenecid	M	10 weeks	5 mg/ml Drinking water	5 weeks Met: day 3–35	*n* = 4–5	Met decreases MPTP-induced loss of TH-positive neurons in SN and reduces levels of inflammatory cytokines
(50)	Swiss Albino Mice	Day 1–5: MPTP: 25 mg/kg + 250 mg/kg probenecid	M	? 22–25 g	500 mg/kg/day Oral gavage	21 days	*n* = 12	Met improves regeneration of MPTP-induced motoric deficits Met decreases MPTP-induced loss of TH-positive neurons in SN and induces BDNF expression
(51)	C57BL/6N	None	F	10 weeks	A: 5 g/kg Diet B: 5 g/l Drinking water	A: 1 month B: 6 months	A: *n* = 20 B: *n* = 4	Met reduces protein levels of phosphorylated α-Synuclein in mouse brains
(52)	C57BL/6J	Day 1: MDMA 20mg/kg	M	3 months	200–400 mg/kg/day i.p. injection	3/8 days Met: 400 mg day 1, 200 mg day 2+3	*n* = 7–12	Met reduces MDMA-induced loss of TH-positive neurons in SN and CPu
**ALZHEIMER'S DISEASE**								
(37)	P301S tau transgenic C57BL/6	Transgenic, tau mutation	m	4 weeks	2 mg/ml Drinking water	4 months	*n* = 12–15	Met reduces Ser262-tau phosphorylation in CX and Hip but increases number of tau inclusions Met induces AMPK phosphorylation and PP2A protein levels and in CX and HIP
(53)	db/db mice (BKS.Cg-m+/+ Leprdb/J)	Transgenic, leptin receptor mutation	M	6 weeks	200 mg/kg/day Oral gavage	6 weeks	*n* = 3–10	Met decreases ^125^I-Ab_1−40_ influx and RAGE expression at the BBB in db/db mice Met ameliorates memory impairments in db/db mice
(54)	Wildtype	None	?	?	5 mg/ml Drinking water	16–24 days	*n* = 6	Met reduces Ser202- and Ser262-tau phosphorylation in mouse brains
(55)	db/db mice	Transgenic, leptin receptor mutation	m	7 weeks	200 mg/kg/day i.p. injection	18 weeks	*n* = 6–11	Met has no effect on spatial learning and memory Met reduces total tau protein levels as well as Ser396 phosphorylated tau in Hip and decreases JNK phosphorylation
(56)	C57BL/6J	None		5 weeks	2 mg/ml Drinking water	1 week	*n* = 4	Met increases BACE-1 and APP protein levels and induces AMPK phosphorylation in mouse brains
**COGNITION**								
(57)	C57BL/6	HFD (60% fat)	M	12 months	1% Diet	6 months	*n* = 16	Met attenuates HFD-induced deficits in motor function and memory
(58)	NIH Swiss mice	HFD (45% fat)	M	6-8 weeks	300 mg/kg BW Drinking water	20 days	*n* = 10	Met does not improve HFD-induced cognitive deficits and has no effect on astrogliosis
(59)	Wistar rats	HFD (45% fat)	M	? 125–150 g	144 mg/kg Diet	10 weeks	*n* = 16–24	Met has no effect on HFD-induces deficits in Matching To Position Test
(60)	Wistar rats	HFD (59.28% fat)	M	6 weeks HFD 13 weeks Met	15 mg/kg 2x/day Gavage feeding	9 weeks HFD 3 weeks Met	*n* = 8	Met reduces HFD-induced memory deficits Met reduces HFD-induced mitochondrial dysfunction
(61)	C57BL/6J	None	M	4/11/22 months	2 mg/ml Drinking water	3 months	*n* = 16–18	Met has no effect or even impairs spatial memory
**HUNTINGTON'S DISEASE AND AMYOTROPHIC LATERAL SCLEROSIS**								
(62)	R6/2- B6CBAF1/J	Transgenic, huntingtin mutation (136-151 CAG repeats)	M/F	5 weeks	2 or 5 mg/ml Drinking water	Around 10 weeks (until death)	*n* = 5–9	Met (2 mg) increases survival time in male mice but has no effect on female mice
(63)	B6SJL-TgNSOD1^G93A^	Transgenic, SOD1 Mutation (G93A)	M/F	5 weeks	0.5 or 2 or 5 mg/ml Drinking water	Around 16 weeks (until death)	*n* = 6–15	Met has negative effect on start of neurological symptoms and disease progression in female mice and has no effect in males

*BBB, Blood Brain Barrier; BW, Body weight; CPu, Caudate Putamen; CX, Cortex; HFD, High-fat diet; Hip, Hippocampus; KO, Knock-Out; MDMA, 3,4-Methylendioxy-N-methylamphetamine; Met, Metformin; MPTP, 1-methyl-4-phenyl-1,2,3,6-tetrahydropyridine; SN, Substantia Nigra; ST, Striatum; TH, Tyrosine Hydroxylase*.

* *If the original article did not contain information about age, body weight was indicated*.

It also seems that metformin is capable of simultaneously having both a negative and positive impact on specific biochemical events within the same disease model. For example, in a P301S tauopathy mouse model, metformin treatment reduced tau phosphorylation but promoted tau aggregation (37). The authors suggest that metformin could be beneficial as a dephosphorylating agent but could promote protein aggregation, the latter being unquestionably the more widely accepted neurodegenerative disease pathology. Similarly, short term metformin treatment again reduced tau phosphorylation but had negative effects since it activates APP and BACE-1 (54, 56). Metformin again seems to have positive effects on reducing total tau and tau phosphorylation at serine 236, whereas the sulfonylurea type diabetes drug glibenclamide performed much better in similar tests (53, 55).

Sex may also influence metformin action, which could complicate the interpretation of animal data. Male rodents are often favored and sometimes the sex of the animals used is either overlooked or omitted entirely. In one metformin study already mentioned, male mice showed impaired cognitive function while female mice were improved after treatment (36).

### Diabetes and dementia: human studies

Changes in cognition have been reported in type 2 diabetes mellitus (T2DM) patients who have not received a diagnosis of dementia and meta-analyses have found moderate but significant deficits across cognitive domains (64–66). T2DM also seems to increase the risk of conversion from mild cognitive impairment to dementia and the conversion from amnestic mild cognitive impairment to AD (67).

Brain imaging studies in T2DM patients have shown a reduction of whole and regional gray matter volume including hippocampal volume when compared to non-diabetics (68, 69). Taken together, the clinical data mostly shows that T2DM patients have an increased likelihood of developing dementia (10, 70–73). The relationship between diabetes and dementia is further strengthened by reports that reversely, AD patients have an increased risk of developing T2DM or impaired glucose tolerance (74–76). Furthermore, *post mortem* brain pathology in AD shows decreased insulin receptors and IGF protein levels, and insulin levels and markers of insulin signaling are altered in the brain (77–80). Hyperglycemia and hyperinsulinemia have also been positively correlated with AD pathology (75, 81, 82). However, it must be stated that the vast majority of neuropathological studies did not find any association between T2DM or indeed glucose levels and extent of AD pathology (83–87) and two studies even suggest a negative association (88, 89).

One explanation for the discrepancy between clinical and neuropathological studies in AD is the influence of vascular pathology. It is now established that concomitant pathologies in the aging brain are rather the rule than the exception (90). The fact that most studies show no association between T2DM and Aβ deposition therefore seems to hint that there is no major effect of T2DM on Alzheimer's pathology. An additional effect of small vessel disease on cognition in patients with T2DM and Alzheimer's pathology could explain the higher likelihood to develop dementia in this group. This implies that even if T2DM does not have a large impact on Alzheimer's pathology the proper management of diabetes in AD is relevant (67, 91). An interplay between T2DM and Alzheimer's beyond vascular pathology should not be disregarded though especially considering evidence on shared pathophysiological features.

### Metformin and dementia: human studies

Results from clinical studies assessing the effect of metformin use on cognitive decline and AD mostly show a positive influence (Table 2). Metformin use is associated with significantly lower risk of cognitive impairment in T2DM (102, 107). The incidence of dementia in general is lower in T2DM patients receiving metformin, sulfonylurea or a combination of both drugs compared to those not receiving oral anti-hyperglycemic agents (96). The risk of developing AD was lower in diabetics receiving metformin than in patients receiving sulfonylurea or thiazolidinediones in two studies (97, 101). However, in a single study, long-term use of metformin for T2DM (though not sulfonylureas or thiazolidinediones) was associated with higher risk of developing AD (103). One informative study used latent class analysis to identify groups of men with T2DM receiving metformin who develop different profiles of comorbidities including dementia. They concluded that the effect of metformin may in fact differ depending on the risk-profile of patient receiving the drug (100).

**Table 2:** Studies evaluating the effect of metformin on incidence and progression of neurodegenerative diseases.

**Study**	**Disease**	**Characteristics**	**Result**
(92)	PD	Retrospective cohort study, 800,000 individuals of whom 61,166 were diabetics, among the latter 41,003 received OAA therapy	Higher PD incidence for patients with T2DM without (HR 2.18) and with (HR 1.30) OAA compared to controls. HR for treatment with metformin alone was lower (0.95) than for sulfonylurea alone (1.57) and the combination showed the lowest HR (0.78)
(93)	PD	Population-based retrospective cohort study with 93,349 T2DM patients receiving metformin (FU of 657,537 patient years) and 8,346 T2DM patients receiving glitazones with or without metformin (FU of 69,338 patient years)	Incidence of PD significantly lower in T2DM receiving glitazones compared to those receiving metformin (HR 0.72), no incident PD in long-term glitazone users who were still taking glitazones
(94)	PD	Population-based retrospective cohort study with 41,362 patients receiving metformin alone, 316,210 patients receiving simvastatin alone, and 52,311 receiving both, metformin and simvastatin	Lower incidence of PD for patients receiving simvastatin alone (HR 0,64) or in combination with metformin (HR 0.74) compared to metformin alone
(95)	PD/Dementia	Retrospective cohort study, 4,651 patients with T2DM with metformin treatment, 4,651 patients with T2DM with metformin treatment; >21,000 person-years of FU	Higher incidence density for PD (HR 2.27), AD (2.13), and VD (2.30) in the metformin group compared to those in the non-metformin group
(96)	Dementia	Retrospective cohort study, 127,209 dementia-free individuals aged ≥50 years, of which 25,939 w/T2DM, 1,864 w/Metformin only, 9,257 w/Sulfonylureas + Metformin	Higher incidence of dementia in T2DM than controls, higher incidence in T2DM wo/ OAA compared to sulfonylurea (HR 0.85), metformin (HR 0.76), or a combination of metformin and sulfonylurea (HR 0.65)
(97)	Dementia	67,731 non-demented, nondiabetic individuals aged ≥65 years observed for 5 years and observation of onset of T2DM, antidiabetic medication and dementia	Increased risk of dementia onset for new-onset T2DM compared to non-T2DM (HR 1.56), risk to develop dementia was higher for thiazolidinedione users than for sulfonylurea and metformin
(98)	Dementia	189,858 individuals with 122,036 receiving metformin and 67,822 not receiving metformin, dementia incidence rate per 1,000 person-years	Patients with diabetes taking metformin had significantly lower dementia incidence rates than those not taking metformin (21.79 vs. 31.58 per 1,000 person-years, *p* < 0.001)
(99)	Dementia	Meta-analysis including 544,093 participants, risk of dementia in patients with T2DM taking insulin sensitizers	Incidence of dementia reduced with metformin (RR 0.79) compared to those not taking insulin sensitizer but not significant (*p* = 0.064)
(100)	Dementia	Latent class analysis to identify subgroups with differential effect of metformin on risk of age related comorbidities in 41,204 men with T2DM with 8,393 metformin users,	Identified 4 latent classes of patients who showed different effects of metformin on risk to develop ARC including dementia
(101)	Dementia	Retrospective cohort study, 17,200 new metformin users vs. 11,440 new sulfonylurea users aged ≥65 years, average FU 5 years	Individuals <75 years of age on metformin had a lower risk to develop dementia than those on sulfonylurea (HR 0.67, 95% CI 0.61–0.73)
(102)	Cognitive impairment	Longitudinal population-based study, 365 persons aged ≥55 years with T2DM of which 204 received metformin	Metformin use inversely associated with cognitive impairment (OR 0.49), longer use associated with lower risk of cognitive impairment
(103)	AD	Retrospective case-control study, 7,086 AD patients and controls were compared for previous use of metformin/other antidiabetic drugs	Higher risk to develop AD for longterm users of metformin (AOR 1.71) but not sulfonylurea (AOR 1.01), thiazolidinediones (AOR 0.87), or insulin (AOR 1.01) compared to non-users
(104)	AD	71,433 patients newly diagnosed with diabetes and 71,311 nondiabetic controls, follow up of up to 11 years	Higher incidence of AD in diabetic patients compared to non-diabetics (0.48 vs. 0.38%), no positive effect of anti-hyperglycemic treatment on risk
(105)	AD	Randomized placebo-controlled crossover study, 20 nondiabetic patients with MCI or mild dementia and AD received mg metformin or placebo for 8 weeks and then switched to the other treatment for 8 weeks	Metformin was measurable in CSF, in pooled post-hoc analysis significant increase in superior and middle orbitofrontal CBF after 8 weeks metformin exposure in ASL-MRI, significant improvement in Trail making test part B, a measure of executive function
(106)	HD	Observational study; 4325 HD patients, of which 121 had T2DM and received metformin	HD patients on metformin fared better in test for verbal and executive function but not in motor assessments

*AD, Alzheimer's disease; AOR, adjusted Odds Ratio; ARC, age related comorbidities; ASL-MRI, Arterial Spin Label Magnetic Resonance Imaging; CBF, Cerebral Blood Flow; FU, Follow-up; HD, Huntington's disease; HR, Hazard ratio; MCI, Mild cognitive impairment; OAA, Oral anti-hyperglycemic agents; PD, Parkinson's disease; T2DM, Type 2 Diabetes; VD, Vascular dementia*.

In an interventional study, Luchsinger and colleagues investigated the effect of metformin given daily for 12 months compared to placebo in overweight patients with amnestic mild cognitive impairment. There was improvement in the selective reminding test in the group receiving metformin but not in other cognitive or biomarker outcomes (108). The results were only marginally significant and there was no correction for multiple measurements which at least suggests that the observed improvement must be confirmed in an independent trial. In another interventional, short-term metformin study, nondiabetic patients with mild cognitive impairment or mild dementia due to AD took metformin or a placebo for 8 weeks. Those taking metformin significantly improved in a measure of executive function but not in other cognitive tests or biomarkers. Again, a multitude of test was performed without correction for multiple testing (105).

Although the majority of data on metformin use in dementia with or without T2DM is generally positive, it should be considered that the effect of metformin likely depends on complex underlying pathological processes and may to some extent be related to an effect on vascular rather than neurodegenerative processes. In some instances, metformin could even exert detrimental effects. Prospective interventional studies have not been able to show convincing evidence of a positive effect of metformin in mild cognitive impairment or mild Alzheimer's dementia but were likely underpowered or of too short duration. More, long-term, controlled metformin studies in large, well-defined dementia cohorts are needed.

## Parkinson's disease

### Background

Parkinson's disease (PD) is a common neurodegenerative disease, affecting over 1% of the population above the age of 60 and around 4% older than 85 (109). PD is characterized by bradykinesia and a combination of rigidity, resting tremor, postural instability, and a large range of non-motor symptoms (110). Like other neurodegenerative diseases, PD is clinically and pathologically heterogeneous, with a large variation in disease onset and progression. Progressive loss of dopamine-containing neurons in the *substantia nigra pars compacta*, located in the mid brain, results in a deficit of dopamine in the striatum (111, 112). Insoluble protein inclusions in neurons, termed Lewy bodies, mainly consisting of aggregated α-synuclein (aSyn) are the main neuropathological hallmark of PD (113). Lewy bodies and protein aggregates are found in multiple brain regions and spread with disease progression (114, 115). The exact biological mechanism leading to aSyn aggregation and neuronal loss remains unknown and currently only the symptoms of PD are treated with dopamine-replacement therapy and in some cases deep brain stimulation.

Approximately 5–15% of PD cases can be attributed to disease-causing genetic variants and around 15% of patients have a first degree relative who is also affected (116). The genetic architecture of PD has been well studied but it is complex. 23 loci and 19 genes have so far been associated with familial forms of PD (117). Like in most neurodegenerative diseases, the majority of cases probably result from a complex interplay of risk modifying genetic variation, environmental factors and chance. Knowledge about the genes involved in PD have allowed insight into the underlying biological pathways. Together with multiple environmental factors and epidemiological data, the genetic data has highlighted several cellular functions and pathways including mitochondrial dysfunction, lysosomal function, inflammation, build-up of aggregation-prone proteins and oxidative stress (118, 119).

Despite large investments in research for neuroprotective compounds for PD, none have so far shown any convincing effects in clinical trials (120).

### Diabetes and PD: animal studies

Rodent studies have shown that there is a link between insulin resistance and development of PD. A high fat diet enhanced vulnerability to the neurotoxins 1-methyl-4-phenyl-1,2,3,6-tetrahydropyridine (MPTP) and 6-hydroxydopamine (6-OHDA) as measured by increased nigrostriatal neurodegeneration and motor deficits (121, 122). Likewise, in an aSyn mouse model of PD a high fat diet led to an accelerated development of locomotor phenotype and earlier onset of neurodegeneration (123).

Insulin resistance can directly interfere with dopamine signaling. Rats fed a high fat diet exhibit impaired nigrostriatal dopamine function (124) and overweight and diabetic mice show degeneration of dopaminergic neurons (125).

### Metformin and PD: animal studies

Only a handful of rodent studies have so far assessed the effects of metformin as a neuroprotective agent in PD. These studies have focused mainly on metformin treatment in combination with acute MPTP induced parkinsonism. Although experimental designs in these studies are quite similar, the results are variable, arguing against differences in modeling as the major cause of metformin's variable effects. However, differences in the dose and duration of MPTP and metformin treatments may be important (Table 1).

Most studies in rodents find that metformin reduces the damaging effect of MPTP on dopaminergic neurons, shown by tyrosine hydroxylase staining (a marker for dopaminergic neurons) in the *substantia nigra pars compacta* (49, 50), striatum (45), or both (48). Two studies suggest that metformin's protective effect may not be specific. A study by Ismaiel and colleagues however, reported that metformin had no protective effect against MPTP-induced neuronal loss in the SN (46) and Bayliss reported no protective effect on dopaminergic neurons in the SN, only in striatum (45).

Metformin's supposed ability to protect against dopaminergic neuronal death induced by the neurotoxin MPTP correlates in three studies to improvements in the motor function of rodents (48–50). Given that both, MPTP and metformin act on complex 1 of the respiratory chain, a mutual influence of the drugs on mitochondrial survival cannot be excluded. It is possible that in these studies metformin primarily reduced the damaging effects of MPTP itself rather than restoring damaged neurons. Therefore, examination of metformin's action in transgenic mouse models rather than acute toxin models of PD might give better insight about its potential. An interesting first hint comes from a study using healthy non-transgenic mice that showed that metformin could reduce aSyn phosphorylation in the brain (51).

### Diabetes and PD: human studies

Studies assessing the risk of developing PD in patients with diabetes have very mixed outcomes (126–132). In one meta-analysis comprising 14 case-control studies, PD risk was decreased in T2DM patients (133). Conversely, Cereda and colleagues describe an increased risk for developing PD in diabetics in four prospective cohort studies but not a higher prevalence of diabetes in in patients with PD in five case-control trials (134). It has to be noted that the case-control trials with the largest populations did consistently show a similar of even higher prevalence of diabetes in PD compared to controls. More recently a meta-analysis including seven population-based cohort studies which also found an increased PD risk in patients with diabetes (135). Taken together the meta-analyses seem to hint toward an increased incidence of PD in T2DM. A potential pitfall is the inclusion of vascular PD in some of the studies. T2DM does contribute to cerebral small vessel disease and therefore non-exclusion of patients with vascular lesions may skew the results toward more patients with T2DM exhibiting signs of parkinsonism. This particular problem was addressed in some studies showing an increased incidence of PD in T2DM and therefore cannot sufficiently explain the discrepancies. From a neuropathological view one study describes an association between increased blood glucose levels with increased risk of Lewy body formation in the *substantia nigra pars compacta* and *locus coeruleus* further supporting a role of T2DM in the pathogenesis of PD (136).

Dementia with Lewy bodies (DLB) and Parkinson's disease dementia (PDD) are common causes of dementia in the elderly (137). PD patients with T2DM are reported to have a greater rate of cognitive decline and lower gray matter and white matter volume, although the group was small (138). PDD patients are more likely to show insulin resistance in an oral glucose-tolerance-test than PD patients without dementia (139). DLB and PDD were less common in patients with diabetes in one study using data from the Swedish Dementia Registry (140), yet T2DM was not significantly associated with PDD in many others (141–144).

### Metformin in PD: human studies

Clinical studies have not looked solely at metformin, but rather metformin compared to, or in combination with other oral anti-hyperglycemic agents (see Table 2). Taken together all the studies look at different medications and are hardly comparable. There is lack of clinical data that suggests a positive effect of metformin on PD risk. Wahlqvist and colleagues tried to determine the effect of sulfonylurea, metformin or a combination of both drugs on the incidence of PD in patients with T2DM. Patients with T2DM receiving sulfonylurea had an increased PD risk compared to those not receiving oral anti-hyperglycemic agents. Metformin alone or in combination with sulfonylurea had no impact, suggesting that metformin might rescue the harmful effect of sulfonylurea (92).

Brakedal and colleagues compared the incidence of PD in patients with T2DM from the Norwegian Prescription Database (NorPD) receiving glitazones with or without metformin or metformin alone. Patients taking glitazones had a significantly lower incidence of PD compared to patients on metformin alone. There was no risk reduction in past users of glitazones, indicating the necessity of long-term or even permanent exposure to glitazones for risk reduction (93). Looking at patients from the NorPD receiving statins, metformin or both showed a lower hazard ratio to develop PD for patients using statins in combination with metformin when compared to metformin alone and the risk was lowest in patients only taking statins (93). The positive effect of statins may come through their anti-inflammatory effect and a reduction of microglial inflammatory response, which has been shown to have a positive effect on striatal dopamine activity (145). The question why metformin seems to have a positive effect when added to sulfonylurea while it has a negative effect when taken together with statins must be addressed. The combination of T2DM and hypercholesterolemia might increase the risk of developing PD more than hypercholesterinemia alone and this risk may not be lowered sufficiently by a combination of statins and metformin. The addition of metformin to sulfonylurea may result in a better control of T2DM than the therapy with sulfonylurea alone thereby reducing effects that promote PD risk. Also, the complex interplay between the different drugs has to be taken into account.

To our knowledge there is no data available on metformin use and disease progression. It is also unclear whether metformin use in individuals without insulin resistance may have a beneficial effect on PD development.

## Other neurodegenerative diseases

There are to our knowledge very few or no reports of metformin studies in other, rarer neurodegenerative diseases such as amyotrophic lateral sclerosis (ALS), Huntington disease (HD), motor neuron disease, or atypical parkinsonian disorders. Here we briefly note relevant studies concerning association with diabetes or use of drugs targeting energy metabolism.

### Amyotrophic lateral sclerosis

ALS is a progressive neurodegenerative disease that is characterized by degeneration of the first and second motor neuron resulting in spasticity and muscle atrophy. Eventually this results in difficulty speaking, swallowing, and breathing and often leads to death within a few years after diagnosis. Neurochemical imbalance and genetic mutations are known to cause ALS, but most cases are sporadic and old-age is an important risk factor. Most drugs available for ALS relieve symptoms only, although the drug riluzole and more recently edaravone have been shown to slow progression of the disease (146, 147).

A protective effect of diabetes in older patients and an increased risk of developing ALS in younger patients with diabetes has been described which is thought to reflect differences in association of ALS with T1DM and T2DM (148, 149). Most studies have shown a decreased risk for developing ALS in patients with T2DM (150, 151). However, other studies reported no significant effect on ALS risk or progression and even a higher risk of developing ALS in T2DM in patients below 65 years of age (152–154). Nutritional status is negatively associated with ALS severity (155) and hypercaloric nutrition has even been suggested as a potential treatment option for ALS. Two trials with the PPAR-γ agonist Pioglitazone (which reduces insulin resistance) (156, 157) have not shown any benefit in disease progression (158).

### Huntington disease

HD is a progressive neurodegenerative disease that causes choreatic movements, psychiatric symptoms, and cognitive decline. The most common form of the disease is of early onset, usually diagnosed around 30–40 years of age. HD is caused by defects in the gene *HTT*, which encodes the protein huntingtin and the mode of inheritance is autosomal dominant. Expansion of CAG repeats in the *HTT* gene leads to the production of an abnormally long version of the huntingtin protein. This results in the protein being broken down by the cell into small, toxic fragments and these protein fragments aggregate and accumulate in neurons causing the disease.

Altered glucose metabolism and increased rates of T2DM have been reported in patients with HD (159, 160) and a high prevalence of T2DM has been reported in a Chinese family with HD (161). However, other studies were not able to identify differences in oral glucose tolerance test or pancreatic tissue between HD patients and controls (162, 163). HD patients with T2DM receiving metformin had better cognitive test results than HD patients without diabetes not taking metformin. This was in stark contrast to the non-HD control group where people with T2DM taking metformin fared worse in the cognitive test compared to non-diabetic controls (106).

## Metformin: mechanism of action in neurodegenerative diseases

**Figure 2:**
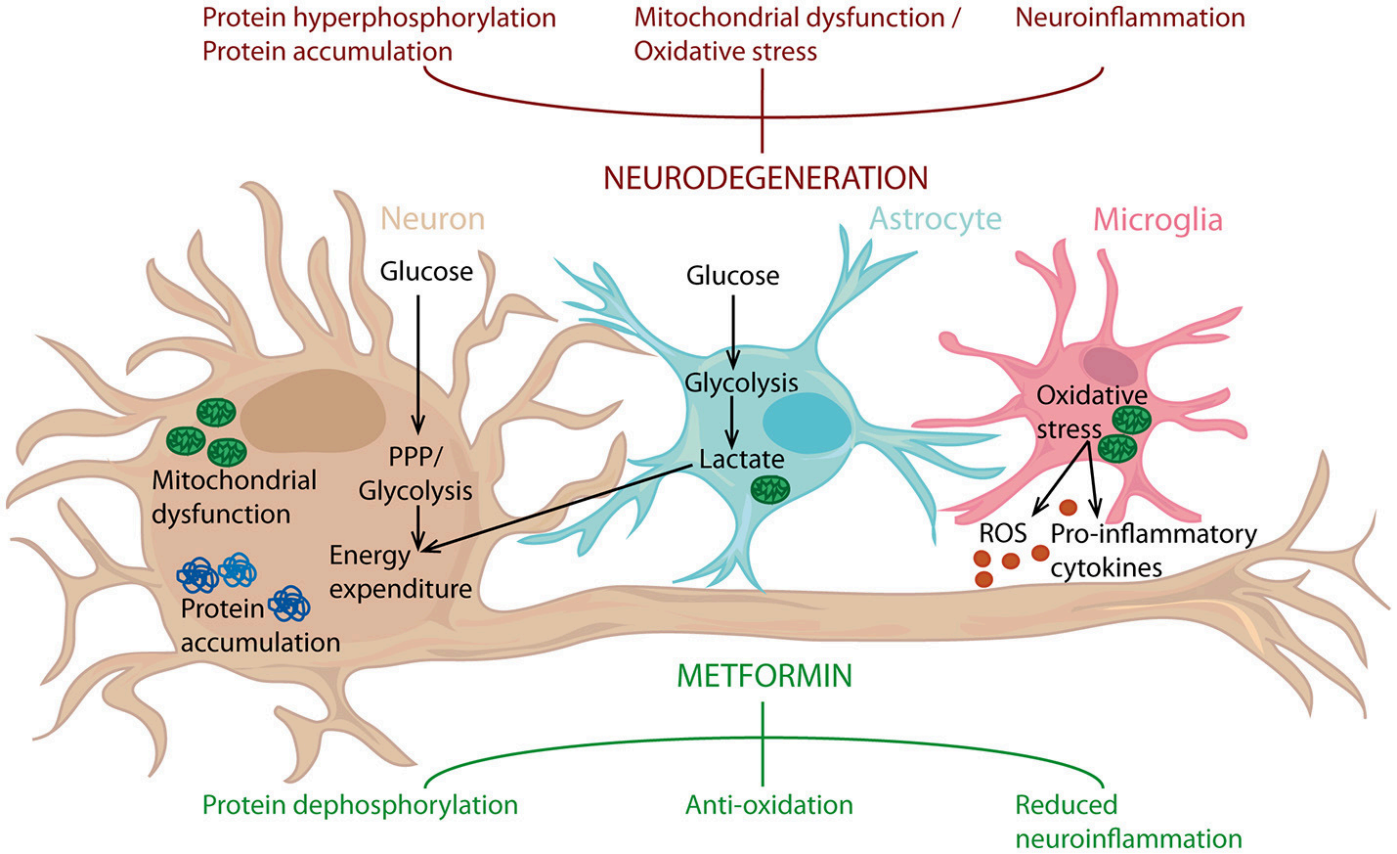
Metformin's potential as a neuroprotective agent. Metformin can counteract protein hyperphosphorylation, oxidative stress and neuroinflammation, processes known to drive neurodegeneration. Metformin can act on neurons, but also targets astrocytes and microglia. Consequently, metformin can influence inflammatory status, along with glucose metabolism in the entire brain and thereby reduce neuroinflammation and act as an antioxidant, leading to protein dephosphorylation. PPP, Pentose phosphate pathway.

The *in vivo* studies conducted so far, regarding the effect of metformin have generated conflicting results. Besides the large differences in study design, these outcomes are probably also due to the many biological pathways influenced by metformin. Here we will discuss some of the biological signaling pathways and biological mechanisms that are the most relevant for metformin's potential as a therapy in neurodegenerative disease (Figure 2).

### Central metabolism and signaling

Central metabolism is tied to the overarching cell signaling pathways involved in proliferation, stress and survival, which are heavily implicated in human diseases including cancer and neurodegeneration. Metformin acts on central metabolism and several major signaling pathways including energy sensing (glucose metabolism and AMPK signaling), mTOR signaling, and inflammatory signaling. Mitochondrial signaling will be addressed separately.

#### Energy sensing and metabolism

The brain constitutes only 2% of the total body mass, but it is one of the main energy-demanding organs in the human body utilizing around 20% of total energy expenditure. Brain cells incorporate (i) the neurons (70–80% of brain energy expenditure) and (ii) glial cells, comprising oligodendrocytes, astrocytes and microglia (accounting for the remaining 20–30% of energy expenditure). The high energy demand of neurons is one of several factors partially explaining the selective vulnerability of certain neuronal subtypes in neurodegenerative diseases. Energy metabolism has long since been thought to play a role in the etiology of neurodegenerative diseases and here we will briefly mention some of the related signaling pathways and biological mechanisms that are relevant for metformin's therapeutic potential in neurodegeneration.

##### AMPK signaling

AMPK is an evolutionarily conserved sensor of cellular energy status. AMPK is activated by increasing AMP levels in conditions of energy deprivation and the enzyme consequently inhibits energy consumption and stimulates catabolic pathways. Activation of AMPK has a wide range of effects, including inhibition of mTor and PI3K-Akt signaling (two important pathways discussed later).

Dysregulation of AMPK is associated with insulin resistance and T2DM (164, 165) and neuroinflammation (166–168). AMPK signaling plays a major role in AD disease progression since AMPK has been shown to regulate both Aβ generation and tau phosphorylation. Inhibition of Aβ production and tau phosphorylation in neuronal cultures is dependent on AMPK activation (169) and activation of AMPK lowers extracellular Aβ accumulation (170). Conversely, in neurons, AMPK activation has been linked to tau phosphorylation as a response to Aβ toxicity (171, 172).

**Figure 3:**
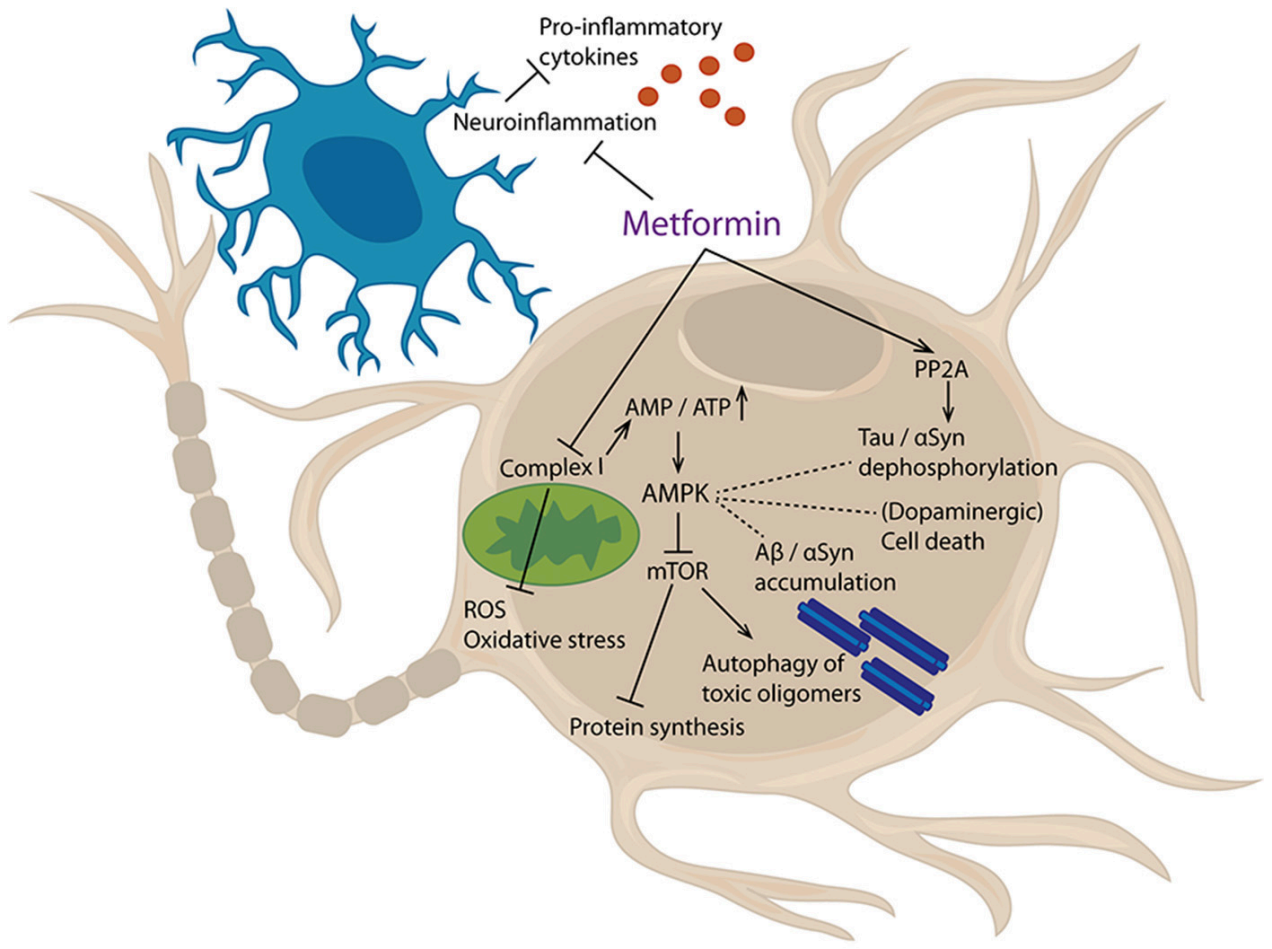
Cellular targets of metformin. Metformin inhibits mitochondrial complex I, thereby increasing AMP/ATP ratio. This lack of energy leads to an activation of AMPK, which, amongst others, inhibits mTor signaling. Furthermore, metformin can activate PP2A and inhibit neuroinflammatory processes. Results of these events are reduced production of pro-inflammatory cytokines and reactive oxygen species (ROS), decreased oxidative stress, inhibition of protein synthesis and augmented autophagy of toxic oligomers. Additionally, protein dephosphorylation, protein aggregation, and cell death are affected.

Metformin inhibits complex I of the electron transport chain needed for mitochondrial respiration, thereby leading to an energy deficit and indirectly activating the AMPK pathway (173–175). Thus, stimulation of AMPK can be seen as a key consequence of metformin administration, explaining many of the known effects of the drug (Figure 3).

However, in the context of AD especially, more studies are needed to understand the complex role of AMPK signaling and the action of metformin. A study conducted in human neuronal stem cells proposed that activation of AMPK via metformin is neuroprotective against Aβ (176) and other *in vitro* studies showed that metformin is able to reduce tau phosphorylation via mTOR/PP2A (Protein phosphates 2A) signaling (54) and that it can reduce molecular pathologies associated with AD (177). An additional level of modulation via AMPK by metformin could come from metformin's ability to reduce BACE1 protein levels in neurons (178). Conversely, metformin was also reported to upregulate BACE1 in neurons and increase the generation of Aβ (179), suggesting detrimental effects of activating AMPK in diseased neurons.

In PD mouse models the AMPK involvement is similarly multifarious. Administration of the neurotoxin MPTP activates AMPK signaling (180). Interestingly, both AMPK overexpression and AMPK inhibition have promoted survival in neurotoxin treated PD models (180) but another study provided evidence for a protective function of AMPK activation in *in vivo* PD models (181).

Overexpression of aSyn in cell culture reduced AMPK activity, while inhibition of AMPK lowered resistance to aSyn toxicity (182). AMPK's subunits α1 and α2 have neuroprotective effects against aSyn toxicity with low but continuous AMPK activity almost completely preventing loss of dopaminergic neurons (183). Accordingly, in rodent PD models dietary metformin influenced neuronal function via AMPK modulated aSyn phosphorylation status (49, 51). However, several other studies point in a different direction. Over-active AMPK promotes aSyn accumulation (184) and hyperactivation of AMPK leads to aSyn binding to the GTPase PIKE-L and dopaminergic cell death (47). These studies show that lower AMPK activity may in fact be beneficial at least in aSyn models of PD.

As is the case in many neurodegenerative diseases, the underlying genetic and biological causes are heterogeneous, often causing multiple pathologies that can overlap across the disease spectrum. The action of metformin primarily via the mitochondria could have numerous and potentially opposite effects on AMPK depending on the amount of involvement and type of mitochondrial signaling in each patient or disease model at any given moment. One important aspect to consider here is that biological pathways are not necessarily fixed in a single state throughout the disease course. Neurons especially have evolved to carefully adapt to energetic needs in order to survive since they are seldom replaced. Sophisticated compensatory mechanisms are initiated for the purpose of mitochondrial rejuvenation and adaption. Such complexity has made modeling neurodegenerative diseases in human neurons challenging and has contributed to the current situation where no causative or “cure all” therapies are available.

##### Glucose metabolism

Glucose is an essential energy substrate needed to sustain neuronal activity and is taken up via glucose transporters expressed in the brain endothelium, astrocytes, and neurons (185). Neurons mostly rely on glucose for energy but utilize ketone bodies during fasting. In contrast to other cell types, in neurons the rate limiting glycolytic enzyme Phosphofructokinase B3 is highly turned over by the proteasome, resulting in the preferential metabolism of glucose via the pentose phosphate pathway (PPP) as opposed to glycolysis (119, 186).

A product of the PPP is the electron donor NADPH, which provides reducing power for anabolic reactions and is crucial for maintaining antioxidant potential. The PPP helps neurons to meet high energy demands, but since neurons are predominantly oxidative, maintaining a fine balance between glycolysis and PPP is essential for counteracting oxidative damage and conserving energy.

Glial cells on the other hand, predominantly metabolize glucose via glycolysis producing lactate and have only very low rates of mitochondrial oxidation. Glia metabolically support axons and lactate can be shuttled across a gradient from glia to neurons (Figure 2) (187, 188). Interestingly, in cell culture, neurons favor lactate over glucose (189) preferring a fast supply of energy over metabolic efficiency. In the human brain, energy demand must be tightly regulated to offset oxidative damage and therefore cell culture and cell culture media effects should be taken into consideration when considering the conflicting data on metformin performed *in situ*.

Inhibiting the PPP and glutathione pathways causes increased levels of oxidative stress and cell death similar to that seen during neurodegeneration (119). Glucose hypometabolism has been shown in PD brains (190) and deregulation of glucose metabolism has been proposed as an early event in the pathogenesis of PD (119). Dunn et al. proposed that dysregulation of glucose metabolism occurs via dysregulation of the PPP, which causes oxidative stress because of less efficient glutathione recycling, and it is this event that underlies the increased levels of oxidative stress observed in PD (119).

Metformin can act in these pathways by slowing oxidative phosphorylation via inhibition of complex I in mitochondria and by inhibiting gluconeogenesis, having the effect of further aiding neurons to reduce their oxidative burden by minimalizing NADH utilization.

##### Insulin signaling

Insulin plays an important role in the brain. It is used as a hormonal signal to control body weight, food uptake, and metabolic homeostasis (191–193). Insulin has also been shown to influence expression of dopamine receptors and concentration of dopamine (194–196). Disturbances in insulin signaling have been implicated in several neurodegenerative diseases including AD, PD, and HD (197–200). Insulin is secreted in response to high blood sugar and acts in different organs including the brain. Activation of the Phosphoinositide-3-kinase (PI3K)—Akt pathway via insulin receptor activation and insulin receptor substrates plays a central role in the metabolic actions of insulin (201). Akt activation regulates proteins such as mTOR, FOXO, and BAD. Overall, Akt has over 100 known substrates and has diverse effects on cellular growth, cell proliferation, glucose uptake, protein synthesis, glycogen synthesis, and apoptosis (202). Akt is inhibited by PP2A (203), PHLPP1/2 (204), and indirectly by PTEN (205). Insulin resistance has been associated with disturbances in signaling up and downstream of Akt (206–208).

Insulin has been administered to patients to try to improve symptoms of neurodegeneration (209, 210) and has been shown to protect cells from Aβ induced death (211–213). The Insulin Degrading Enzyme (IDE), originally found to play an important role in insulin turnover (214) is involved in Aβ degradation. IDE can degrade secreted Aβ from neurons and microglia and mediate its clearance (215). Furthermore, IDE hypofunction can contribute to *in vivo* Aβ accumulation (216). In hippocampi of ApoE4 carriers reduced expression levels of IDE have been measured (217) and genetic differences in IDE expression and activity have been suggested to be involved in AD development (218–221). Reduced levels of IDE in liver and brain have been correlated with aging (222) and IDE can counteract damage from oxidative stress, suggesting a neuroprotective role (223–226).

Metformin lowers blood glucose levels through inhibition gluconeogenesis in the liver via AMPK (227, 228). AMPK inhibits PI3K/Akt signaling, the crucial pathway downstream of the insulin and IGF1 receptors (229). Metformin has also been shown to act on insulin signaling independently of AMPK. Metformin is reported to downregulate expression of insulin and IGF-1 receptors (230, 231) and reduces phosphorylation of insulin receptors (232) including IRS-1 (230, 233).

Both acute and chronic metformin administration has been found to increase levels of GLP-1, an incretin known to induce insulin secretion, in humans and mice (234–236). Very recently a randomized, double-blind, placebo-controlled trial for PD showed that a GLP-1 agonist had positive effects on motor symptoms in PD (237), generating a new potential mechanism for metformin action in neurodegeneration.

##### mTOR Signaling

mTOR signaling is a highly conserved and central signaling pathway integrating upstream signals such as nutrient and redox status and then controlling downstream processes such as cellular growth, motility, survival, and death (238). The mTor pathway is crucial for regulating mitochondrial biogenesis and autophagy, two processes that are defective in many neurodegenerative diseases.

**Figure 4:**
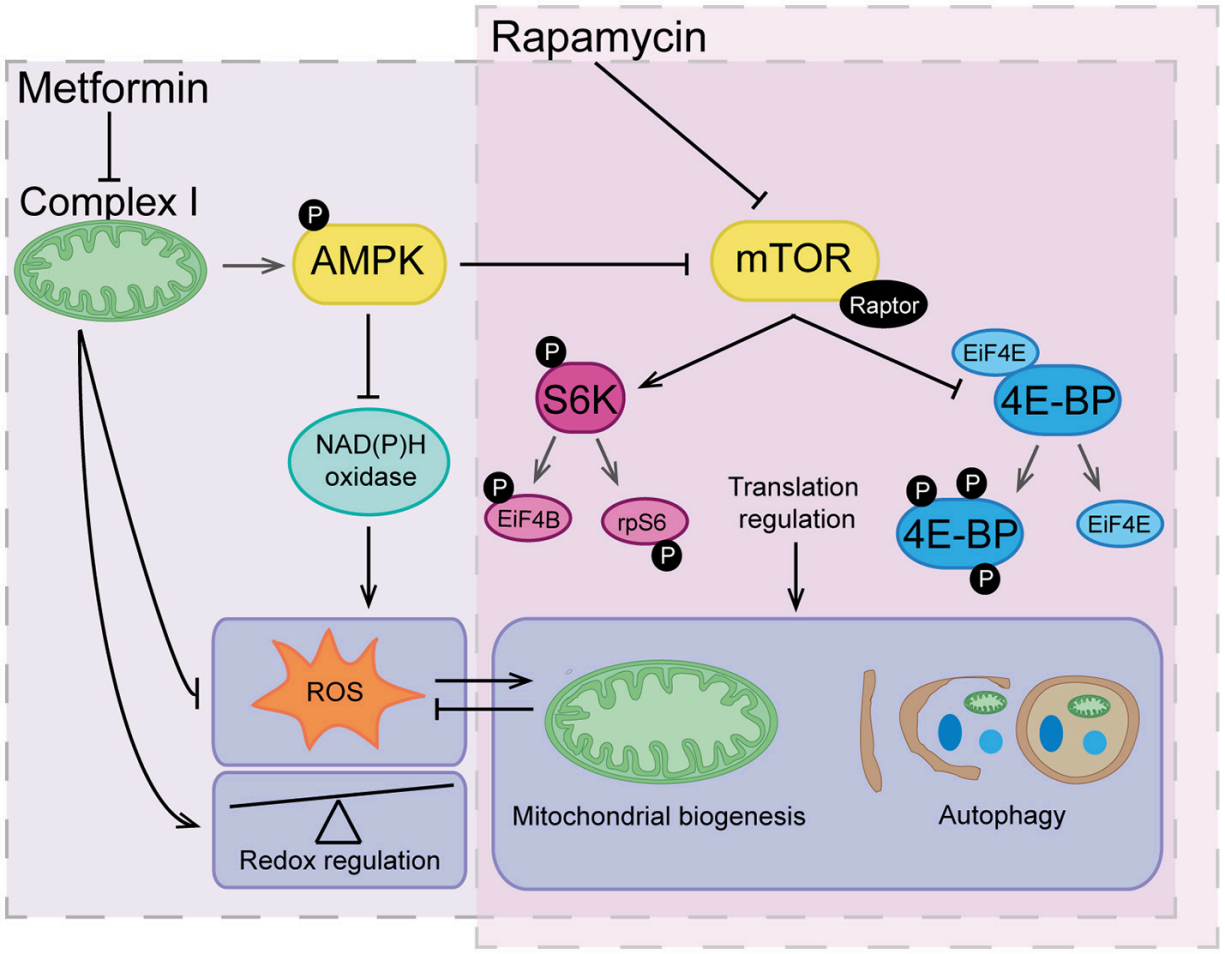
The overlapping actions of metformin and rapamycin. Rapamycin acts by directly inhibiting mTOR and therefore translation regulation, which has a major influence of highly regulated processes such as mitochondrial biogenesis and autophagy. Metformin acts indirectly on the mTOR pathway through inhibition of complex I and activation of AMPK signaling. Metformin also reduces reactive oxygen species (ROS) via inhibitory action on complex I and NAD(P)H oxidase having an overall effect as a redox regulator. Downstream of metformin action, low level ROS can indirectly trigger signals for mitochondrial biogenesis and turnover of organelles and proteins via autophagy. Vice versa, maintenance of healthy mitochondrial networks involving autophagy and mitochondrial biogenesis further reduces build-up of damaging levels of ROS.

mTOR is a serine/threonine protein kinase, composed of the protein complexes mTORC1 and mTORC2. mTOR signaling is targeted by the PI3K/Akt pathway, the key insulin signaling pathway (239, 240). Both PTEN (241, 242) and AMPK (243, 244) suppress mTor signaling and rapamycin is a well-studied inhibitor of mTORC1 (245–247). Although mTor signaling influences many downstream events, the most important mechanism of action is through the phosphorylation and activation of S6K1 and 4E-BP1 and subsequent control of RNA translation (238) (Figure 4). Interestingly, deficiency in mTor signaling has been implicated with insulin resistance and diabetes. Nutrient dependent stimulation of S6K1 can induce insulin resistance (248, 249) and S6K1 deficiency protects against high fat diet-induced insulin resistance (250).

The mTOR inhibitor rapamycin suppresses neurodegeneration phenotypes in mice (251) and protects against MPTP-induced loss of dopaminergic neurons (252). Rapamycin also prevents the development of dyskinesia without affecting the therapeutic efficacy of L-DOPA and thus, the mTORC1 signaling cascade represents a promising target for the design of anti-Parkinsonian therapies (253).

Elevated mTOR signaling has been found in AD patients and is linked to diabetes and aging (254, 255). Rapamycin abolishes cognitive deficits and reduces Aβ levels in a mouse model of AD (256). It also ameliorates AD-related phenotypes by restoring hippocampal gene expression signatures (257). Importantly, mTor regulates tau phosphorylation and degradation (258), making this pathway an interesting target for the treatment of tauopathies.

If we compare the therapeutic potential of metformin, a well-known inhibitor of mTOR signaling through activation of AMPK (259) to that of rapamycin, which is more widely accepted in the field, the obvious difference is that metformin action on mTOR is relatively indirect. Rapamycin forms a complex with the FKBP12 binding protein which binds and specifically alters mTORC1. Metformin acts on the mTOR pathway indirectly via multiple routes. The AMPK independent routes include inhibition of transcription factors (260), the PI3K/AKT pathway (261), and induction of REDD (262). In direct contrast to this, one study has shown that metformin can directly inhibit mTORC1 and is dependent on Rag GTPases not AMPK (263). These data support the view that metformin has more than one direct target and is likely to have many more indirect targets, thus explaining why the use of metformin and a research tool or treatment is less accepted than rapamycin.

Nevertheless, the mTOR pathway links several biological pathways underlying neurodegenerative diseases and therefore the ability of metformin to inhibit this signaling cascade endorses the argument that more mechanistic work using metformin and its inclusion in clinical trials should be positively considered.

### Inflammation

Neuroinflammation is considered a major driving force in the progression of neurodegenerative diseases and the triggering of innate immune mechanisms is emerging as a crucial component in disease pathogenesis. Microglia and other cell types in the brain can be activated in response to misfolded proteins or aberrantly localized nucleic acids. This diverts microglia from their physiological and beneficial functions, and leads to their sustained release of pro-inflammatory mediators (264).

Intake of non-steroidal anti-inflammatory drugs (NSAIDs) has been reported to decrease incidence of AD later in life (265, 266) and activated microglia are found in brains of AD patients (267, 268).

In AD, an integrated network-based approach identified gene perturbations associated with innate immune pathways and microglia cells in late onset forms of the disease (269). AD patients show increased expression of inducible nitric oxide synthase (iNOS, a product of neuroinflammation) in neurons and glia, leading to augmented nitric oxide production (270, 271). Activated microglia can further induce tau phosphorylation in primary mouse neurons, activating IL1β receptor and p38 MAPK stress signaling (272).

In PD, patients show increased numbers of activated microglia and astrocytes (273, 274) and microglia activation has been associated with disease progression (273, 275, 276). aSyn has been found to activate microglia, enhancing neurotoxicity (277). Activation of microglia increases nitration of aSyn, resulting in neuronal cell death (278).

Immune signaling triggers transcriptional events, but also changes in metabolic flux, redox balance, and metabolite balance via mitochondria (279). Mitochondrial dysfunction is associated with neuroinflammation (280) and even moderate mitochondrial DNA stress can trigger antiviral signaling (281).

Metformin reduces general inflammation parameters and inhibits NF-κB signaling as well as proinflammatory cytokines in different cell types (282–285), suggesting that metformin could protect against neuroinflammation. Interestingly, in two MPTP-induced PD mouse models, metformin reduced levels of the microglia marker Iba1 as well as the pro-inflammatory cytokines TNFα, Il-1β, IL-6, and iNOS in the *substantia nigra pars compacta* (46, 49). Here, more studies are needed but metformin seems to have a wholly positive effect against general inflammation. Neuroinflammation is a recognized event associated with neurodegenerative diseases and therefore metformin could be both a useful tool and therapy.

### Mitochondria

Mitochondria are crucial organelles that produce energy and perform a plethora of other functions needed for central metabolism and cell signaling. Mitochondrial dysfunction is a phenomenon that traverses all neurodegenerative diseases and forms the basis of β-cell dysfunction in T2DM (286). One important aspect of mitochondrial dysfunction in neurological disease is that the need for tightly controlled energy metabolism in neurons can partially explain some of the vulnerabilities involved in their demise.

#### Parkinson's disease

In PD, the link between mitochondrial dysfunction and disease has been proven by the identification of environmental factors and disease genes which critically affect mitochondria. The outcome has been a large body of work depicting the role of mitochondrial dysfunction in PD, yet the exact mechanisms underlying sporadic forms of PD are less defined.

Loss of function mutations in PINK1 or parkin cause PD (287–290) as a result of mitochondrial dysfunction and this has been elucidated *in vitro* (291, 292) and *in vivo* (293–296). PINK1 and parkin act in a pathway that is important for mitophagy (removal of damaged mitochondria via the lysosome) induced by mitochondrial depolarization. Here, PINK1 functions upstream of parkin (295, 297). Upon mitochondrial damage, PINK1 accumulates on the mitochondrial surface and selectively recruits parkin to mitochondria (298, 299). Mitochondrial substrates are ubiquitinylated, leading to the removal of damaged mitochondria. PINK1 is now known to be a ubiquitin kinase (300) but may have other functions yet unknown. For example, PINK1 is not required for basal mitophagy *in vivo* (301, 302) and has been proposed to regulate complex I (303), mitochondrial dynamics (304), mitochondrial proteostasis (305), and mitochondrial metabolism via TRAP1 (306, 307).

PINK1 and parkin are upregulated under metabolic stress in the vessel walls of obese and diabetic mice and have a protective action by limiting reactive oxygen species (ROS) production and mitochondrial dysfunction (308). In a diabetic mouse model, PINK1 expression in the hippocampus was in this case reduced following hydrogen peroxide treatment (309), further suggesting that PINK1 plays a role as a stress sensor and functions accordingly in diverse ways. PINK1 is generally associated with neuroprotection since loss of function causes PD, but because PINK1 is normally highly turned over at the mitochondrial outer membrane and therefore overexpression and/or altered expression might also induce unwanted downstream events. In one study, PINK1 overexpression restrained MAPK and ROS signaling and mitigated insulin resistance in cell models (310). Conversely, PINK1 loss corrupts function of islet and β-cells causing impaired glucose uptake and increased levels of plasma insulin (311). Further evidence that PD proteins play important roles in energy metabolism is a study showing that TP53INP1 deficient cells (TP53INP1 is a susceptibility locus in T2DM) causes an increase in ROS that impairs mitophagy via the PINK1-parkin pathway (312).

Parkinson's disease mutations in aSyn are associated with several cellular defects, including reduced mitochondrial integrity and function. Recent work has identified a highly neurotoxic aSyn species which induces mitochondrial damage and mitophagy in the human and animal brain (313). However, the consequences of these mitochondrial changes for bioenergetic functions remains somewhat undefined. Interestingly, aSyn toxicity is mitigated by TRAP1 (314), a mitochondrial ATPase that has been linked to metformin.

In this pathway, TRAP1 and the mitochondrial serine protease HtrA2 are both targets of the PD protein PINK1 (305, 306). HtrA2 and TRAP1 genetic variants have been found in PD patients (307, 315) but the mutations are rare and a controversial topic (316–318). Regardless of the genetic contribution to disease, TRAP1 at least appears to play an important regulatory role in mitochondria that is relevant for the fine tuning of energy metabolism. TRAP1 is well studied in cancer since TRAP1 expression is tightly regulated in tumor cells (319), TRAP1 acts as a metabolic switch (320) by targeting and inhibiting succinate dehydrogenase (321), which is important for metabolic re-purposing and inflammatory responses (322).

In ovarian cancer where TRAP1 expression was altered, metformin was effective in rendering the tumor sensitive to chemotherapy (323), suggesting that metformin might be relevant to TRAP1 mediated signaling. On this basis, metformin was then used to successfully rescue mitochondrial dysfunction in a TRAP1 cell model of PD (307). In a healthy person, fine tuning of mitochondrial energy usage via the PINK1-HtrA2-TRAP1 pathway and other regulatory mechanisms may allow cells to conserve energy and reduce oxidative burden. Metformin's ability to mimic this fine tuning role *in vitro* was beneficial in one model of sporadic PD (307). However, there are still a lot of questions that remain unanswered such as whether metformin is beneficial in non-diseased neurons, aging neurons and other forms of familial and sporadic PD. One question is whether metformin could specifically target energetic deficits in the dopaminergic neurons of the *substantia nigra pars compacta*. The question is not yet answered because selective vulnerability is still not yet fully understood. We can speculate that oxidative or metabolic burden over time could contribute to making these cells especially vulnerable. Many redox reactions happen in mitochondria as a result of mitochondrial activity. Neurons in comparison to many other cell types have a high energy demand and because of the autonomous pacemaking in dopaminergic neurons of the *substantia nigra* (324), these cells are thought to have a higher oxidative burden. The metabolism of dopamine itself is highly oxidative and can form several toxic species. Therefore, if metformin can mildly reduce the oxidative burden at the mitochondria without interfering with normal redox signaling and stimulate autophagy and other processes which can become less effective over time, it could be seen as a very useful drug to counteract neurodegenerative diseases. Neurons have a sophisticated and unique line of quality control defenses which allow them to compensate for stress and survive against all odds because once they die, inflammation often ensues and they are seldom replaced. It just depends whether metformin treatment could be used to intervene at the right time to not interfere with necessary compensatory responses, rather enhance them.

#### Alzheimer's disease

The exact mitochondrial events leading to AD are less defined than in PD, yet aging is still the greatest known risk factor. Energy metabolism and mitochondrial dysfunction have been proposed as a primary event in mechanisms underlying AD such as synaptic degeneration, Aβ deposition and formation of neurofibrillary tangles (325). There is a vast amount of evidence that mitochondrial dysfunction occurs after the early cellular events in AD and can contribute to the advancement of further degeneration, but it is often unclear whether mitochondrial dysfunction is indeed just a secondary event or whether it might be involved in primary pathogenesis. For example, in the case of tau, abnormal tau triggers oxidative stress and mitochondrial defects such as mitochondrial depolarization, impaired mitochondrial complex activities and reduced energy output (326, 327). Tau also localizes to the microtubules, the tracks on which mitochondria move along with the help of adapter proteins and defective mitochondrial movement has been shown in several models of AD (328, 329).

There is also evidence that mitochondrial metabolism is altered in AD brains (reviewed in (330)). The tricarboxylic acid (TCA) cycle enzymes pyruvate dehydrogenase, isocitrate dehydrogenase, and alpha-ketoglutarate dehydrogenase are affected in AD brain tissue and in patient-derived fibroblasts (331). Changes in these checkpoint TCA cycle enzymes are associated with metabolic re-wiring often in response to stress and redox changes. In addition to matrix enzymes, deficiencies in oxidative phosphorylation (OXPHOS) have been reported [reviewed in (332)].

In AD research, there are few mechanistic models for mitochondrial dysfunction, mainly due to the fact that there are no mitochondrial causative genes for AD. The mitochondrial mechanism of metformin action in dementia and AD is likely similar in PD, in that metformin can act on mitochondrial quality control via mitochondrial biogenesis and energy conservation.

#### The complex I paradox

Many of metformin's actions are thought to be an indirect result of complex I inhibition. The exact inhibitory mechanism of metformin on complex I is not fully understood. The inhibitory mechanisms of other complex I inhibitors such as MPTP and rotenone are better known in terms of binding site and mechanism of toxicity, especially in disease.

Complex I deficiency has long since been associated with mitochondrial dysfunction and Parkinson's disease risk [for a review see (333)]. Complex I deficiencies have also been reported in AD, HD and ALS (332). The neurotoxins MPTP and rotenone inhibit complex I and generate toxic levels of ROS, which leads to neuronal cell death. It is possible that sub-lethal concentrations of mitochondrial inhibitors that do not generate ROS (or generate less ROS) could be beneficial but little is known.

It is generally accepted that metformin does not generate dangerous levels of ROS. Pharmacologically reducing oxidative phosphorylation and thus the oxidative burden (at the right moment) without generating too much ROS is certainly a challenge. We found that sub lethal concentrations of the specific mitochondrial complex V inhibitor oligomycin, could rescue mitochondrial dysfunction in a TRAP1 deficient PD model to a similar extent as metformin (307) but since metformin is an approved compound for human consumption, we followed up the protective effects of metformin only. It might be interesting to assess the potential neuroprotective action and toxicity with a titration of several respiratory chain inhibitors that act at different sites. For example, the mitochondrial complex III inhibitor, antimycin A is known to generate large amounts of ROS (334), but oligomycin and other disrupters of the respiratory chain have been shown to generate little or no ROS (335).

#### Aging

The main hallmarks of aging set out by Lopez-Otin are genomic instability, telomere attrition, epigenetic alterations, loss of proteostasis, deregulated nutrient sensing, mitochondrial dysfunction, cellular senescence, stem cell exhaustion, and altered intercellular communication (336). All of these hallmarks in one way or another are associated with the pathogenesis of neurodegenerative diseases. Here we will focus attention on some specific aspects relating to these hallmarks that could be the most relevant to metformin's mechanism of action at the mitochondria.

##### Mitonuclear protein imbalance

Human mitochondrial DNA (mtDNA) is bound inside nucleoid bundles, has a high copy number, is inherited maternally and has a high mutation rate (337). Mitochondrial damage and/or depletion induces stress-signaling and adaptive metabolic responses. MtDNA instability is a physiologically relevant stress observed in many human diseases and aging (281). Mitonuclear protein imbalance, is a stoichiometric imbalance between nuclear and mitochondrially encoded proteins and is activated as a key longevity response across many species (338). Alterations to mtDNA are directly linked to respiratory chain dysfunction in sporadic PD patients and it has been shown that complex I is initially affected followed next by complex IV (339). It is thought that imbalance in the stoichiometry between mitochondrially translated proteins and nuclear encoded ones is both a cellular signal and marker of mitochondrial adaption. The mTOR inhibitor rapamycin is used as a tool to initiate mitonuclear protein imbalance (338) and metformin is capable of modulating mitonuclear protein imbalance in human cells (307).

##### Oxidant stress and senescence

The production of reactive species is usually balanced by the cell's antioxidant defenses. An imbalance in the amount of ROS to antioxidant defense results in oxidative stress and can cause damage to proteins, lipids and nucleotides.

Mitochondria are a major source of ROS due to oxygen use in energy production through the electron transport chain. Electrons leak while they are being transferred along the complexes of the electron transport chain. Leaked electrons can react with molecular oxygen to form superoxide radicals. Superoxide can react with Mn-SOD to form hydrogen peroxide, a ROS and a signaling molecule. Hydrogen peroxide is either broken down to form water or it can react with metals to form the highly reactive hydroxyl radical. In mitochondria the main leakage sites are at the transfer of four electrons to oxygen at Complex IV, but also complex I, complex III and via certain dehydrogenases of the TCA cycle in the mitochondrial matrix. Consequences of oxidative stress include proliferation, adaption, damage, senescence, or death depending on the cell type and severity [for review see (340, 341)]. Neurons need to counteract a great deal of ROS because of high energy bursts and catecholamine neurotransmitter metabolism.

Oxidative damage is a major contributor to neurodegenerative diseases [for a review see (342)]. Both oxidative stress and oxidative damage can lead to stress adaption. One such adaptive mechanism in mitochondria might be finely-tuned inhibition of respiratory complexes or mitochondrial uncoupling via uncoupling proteins. There is mounting evidence that mitochondrial uncoupling proteins are neuroprotective [for a review see (343)]. Cellular senescence can occur when adaptive responses are unable to properly protect key molecules from damage to the extent that a cell can no longer divide.

The PD protein DJ-1 provides a link between neurodegeneration and energy metabolism. DJ-1 acts as a chaperone and protease to stabilize mitochondria and protect cells from oxidative stress (344). Several other cellular functions have been attributed to DJ-1, including; binding of Ras as a transcriptional co-activator (345), negative regulation of the phosphoinositide-3-kinase (PI3K)/AKT signaling cascade through inhibition of PTEN (309, 346, 347), chaperone function (348, 349), and RNA binding (350). Although controversial, DJ-1 has also been claimed to have glyoxalase (351) and deglycase (352) enzyme activities (353). DJ-1 also influences insulin secretion as well as β cell viability in the pancreas and DJ-1 knockout mice show increased ROS levels in islet cells, impaired glucose tolerance and decreased insulin secretion (354).

## Gaps in the research

Trials using metformin to treat or protect against neurodegenerative diseases in humans and animals have produced mostly conflicting results. The data shows either positive, no or even detrimental effects of metformin on neurodegenerative processes in cell cultures, animals and humans.

The outcome may depend on the species, cell type or underlying metabolic state. Two promising research areas however are neuroinflammation and aging, yet more work is needed. Very few studies have looked directly at the role of metformin in neuroinflammation, but since this is a growing research focus in the field, more metformin studies may arise. The exact role of metformin in aging is a question that needs to be at least partly understood before we can progress further in understanding its potential to treat neurodegenerative diseases. A major hurdle to this is the lack of good human aging models mainly *in vitro* but also *in vivo*.

Another gap in the knowledge is whether there are potential adverse effects of metformin use in non-diabetics. For example, it has been well documented that long term metformin use leads to vitamin B12 deficiency (355). Vitamin B12 and folate are needed for transmethylation and hydroxylation reactions from amino acids that are crucial for neurotransmitter biosynthesis. How much influence could this have in a patient with disturbed neurotransmitter metabolism and/or those receiving other medications.

## The therapeutic potential of metformin: feasibility

There are several reasons why the use of metformin to treat neurodegeneration could bring about doubt from clinicians and scientists when considering its potential as a therapy or as a research tool. The main point being that metformin seems to be acting on a plethora of biological pathways, and therefore it is very difficult to pin down mechanisms. The second point is the controversial subject of “anti-aging” drugs in general. Since we know very little about the biological underpinnings of aging and know even less about how to efficiently model it in the laboratory, the promotion of an “anti-aging” drug often conjures up more questions than it answers. Then there are several other sticking points among researchers, one being the fact that metformin acts by inhibiting mitochondrial respiration, the exact effect that has been shown by years of research in the Parkinson's disease field to in fact contribute development of disease.

In direct contrast, there are several arguments for metformin being a feasible and useful drug. Firstly, glucose metabolism is of central importance to neuronal redox status, therefore to the long-term survival of neurons. Secondly, as a population we are increasingly insulin resistant and therefore metformin is particularly apt. Metformin is a cheap and safe drug with few side effects and therefore more work *in vitro, in vivo* and in trials will be welcomed.

Nir Barzilai, the director of the Institute for Aging Research at the Albert Einstein College of Medicine suggests that metformin and other related drugs can extend our years of healthy, disease-free living by decades (356). Other scientists have not specifically mentioned metformin but in his 2005 book on mitochondria, Nick Lane suggests that if we live longer to rid ourselves of diseases of old age we need more mitochondria and perhaps a more refined free-radical detection system (357). Whether metformin is capable of modifying the detection system at the right physiological moment without deleterious effects is at least an exciting possibility.

## Future developments in the field

There is potential that metformin could be beneficial in the task of counteracting aging and clinical studies imply that metformin may have positive effects on cognition in T2DM patients. A better understanding of how metformin works will help researchers in the neurodegeneration field to successfully design future research and trials. Upcoming studies such as TAME (358) will help in this respect.

The anti-aging effects of metformin could be summarized by its ability to interfere with the multistage process of energy production without producing damaging amounts of ROS. This action alone could be seen as neuroprotective and metformin may further protect by activating other biological pathways. For example, slowing mitochondrial energy production can also trigger a cascade of signaling events in the liver that result in reduced glucose and insulin. The key role of insulin in nutrient sensing which balances growth and proliferation with life-extending conservation, makes metformin an interesting drug. The field of aging research is growing and *in vivo* and *in vitro* aging models are advancing.

Probably due to the complexity of metformin action, this drug will not likely serve as a potential treatment for neurodegenerative diseases on the current stage because much more work is needed to understand the role of aging in different neurodegenerative disease forms. The greatest value of metformin today might lie in its potential to help decipher those mechanisms underlying neurodegeneration.

## Author contributions

CR, GM, and JF contributed equally to the writing and the editing of the manuscript. All authors approved the final version and submission of this article.

### Conflict of interest statement

The authors declare that the research was conducted in the absence of any commercial or financial relationships that could be construed as a potential conflict of interest.
